# Health-related quality of life and associated factors among Onchocerciasis patients in southeast Nigeria: A cross-sectional comparative study

**DOI:** 10.1371/journal.pntd.0010182

**Published:** 2022-02-09

**Authors:** Adah E. Otache, Ifeyinwa L. Ezenwosu, Edmund N. Ossai, Elias C. Aniwada, Emmanuel A. Nwobi, Benjamin SC. Uzochukwu

**Affiliations:** 1 Department of Community Medicine, University of Nigeria Teaching Hospital, Enugu State, Nigeria; 2 Department of Community Medicine, College of Health Sciences, Ebonyi State University, Abakaliki, Nigeria; Washington University School of Medicine, UNITED STATES

## Abstract

**Introduction:**

Onchocerciasis, a neglected tropical disease of public health importance, causes chronic morbidity and severe disability that may impact on health-related quality of life (HRQoL) of the infected people. This study assessed the HRQoL and associated factors among onchocerciasis patients in southeast Nigeria.

**Methods:**

This was a community-based cross-sectional comparative study. Using a multistage sampling technique, 340 onchocerciasis patients were selected and matched for age and gender with the healthy population in the same neighbourhood. The respondents were interviewed using the short-form-36 (SF-36) questionnaire to determine their HRQoL. WHO Disability Assessment Schedule 2.0 tool (WHODAS 2.0) was used to assess disability in persons with onchocerciasis. Means were compared with independent student t-test while Chi-square test was used to compare proportions. Also, correlation analysis and logistic regression were used in the analyses.

**Results:**

A significantly lower proportion of people living with onchocerciasis had a good quality of life when compared with the healthy subjects (69.4% vs 93.5%, p<0.001). Also, an inverse relationship was seen between disability and quality of life in the onchocerciasis group (r = -0.647, p<0.001). Predictors of poor quality of life among respondents with onchocerciasis were: respondents aged ≥48 years (AOR = 2.5, 95% CI: 1.4–5.0), those with some disability associated with onchocerciasis (AOR = 3.33, 95%CI: 1.4–5.0) and respondents who perceived themselves as a burden to people (AOR = 10, 95%CI: 2.5–20).

**Conclusion:**

Onchocerciasis impacted negatively on HRQoL of persons with onchocerciasis when compared with the healthy population. The quality of life of persons affected with onchocerciasis reduces with increasing disability. There is the need to increase community awareness on onchocerciasis to ensure early diagnosis and prompt treatment as this will reduce disability among those affected with the disease thus enhancing their HRQoL.

## Author summary

Onchocerciasis is a neglected tropical disease caused by *Onchocerca volvulus*, a filarial nematode that is transmitted through the bite of infected blackflies of the genus *Simulium*. The disease manifests either as ocular or as dermal onchocerciasis with several symptoms including itching, nodules, skin thickening, visual impairment and blindness. The disease is still of public health concern especially in the study area where it is endemic and causes debilitating symptoms. This study assessed the Health-related quality of life and associated factors among persons with onchocerciasis. The findings of this study showed that the quality of life of onchocerciasis patients was lower than the healthy population. Also, an increased level of disability among the onchocerciasis patients leads to a reduction in their quality of life which worsens with increasing age. Health professionals should be aware of these findings because it will help them implement and continue community awareness programs on onchocerciasis for early detection and treatment to reduce disability and enhance health-related quality of life.

## Introduction

Onchocerciasis also known as River blindness, belongs to a group of diseases called neglected tropical diseases (NTDs) and is caused by *Onchocerca volvulus*, a filarial nematode for which humans are the only definitive hosts. [1] The disease is transmitted by the bite of an infected black fly (*Simulium spp*.) that breeds in fast-flowing water bodies, and transmission occurs within limited areas of Africa, Central and South America, and the Arabian Peninsula. [2] The endemic areas in Africa are the savanna and tropical forest zones south of the Sahara Desert of which since 1973 to date, several control programmes have been ongoing in those areas to eliminate the disease. [2]

Onchocerciasis is a parasitic infection of public health importance. [3] Its prevalence and the magnitude of its associated social and economic effects vary widely in different geographical areas where the disease occurs. Approximately 37 million people are living with onchocerciasis infection worldwide with more than 99% of the infected people living in 31 African countries and 120 million people at risk of contracting the onchocerciasis infection in the Africa regions. [3,4] Furthermore, in Nigeria, 7–10 million are infected with the disease and about 50 million people are at risk of being infected with the disease. [5] Also, Nigeria accounts for nearly 40% of the world’s prevalence of onchocerciasis. [6]

The disease can cause skin impairment and blindness which manifests clinically as onchodermatitis, pruritus, depigmentation of the skin or leopard skin, subcutaneous nodules, hanging groin, lymphoadenopathy, onchocerciasis associated epilepsy and temporary vision loss to blindness. [7] It is the second most common cause of preventable blindness in the world and every year an estimated 40,000 new blind cases are due to the disease. [8,9] People with onchocerciasis experience reduced life expectancy, social stigma, low productivity and high health costs which lead to loss of income, poor sleep, worry, low self-esteem and emotional pain thus affecting their health-related quality of life. [3]

Health-related quality of life (HRQoL) is an assessment of health state which reflects the physical, psychological, social and emotional well-being of patients. [10] It takes into account not only the disease but also the psychological and social impact of the disease. [10] Onchocerciasis like other NTDs has the potential to increasingly affect the HRQoL measures of the individuals differentially depending on the degree of lesion or disability when compared to the normal population. [11] Understanding the different dimensions of health-related quality of life that affect people with onchocerciasis and factors that affect it, will help health professionals to go beyond addressing the clinical presentations to taking care of the psychological and social wellbeing of the infected persons. [12] Also, it will guide the policymakers in formulating and implementing policies or interventions to improve their quality of life. [11] Several studies have been done on health-related quality of life in people with other chronic conditions [13] but limited studies exist on the health-related quality of life in people living with onchocerciasis. Furthermore, onchocerciasis is a severely debilitating and disfiguring disease that impedes the socio-economic and psychological wellbeing of infected persons especially in areas of high endemicity which includes Southeast Nigeria. [14] Therefore, this study compared the health-related quality of life of people with onchocerciasis to the healthy population and the various factors affecting their quality of life.

## Methods and materials

### Ethics statement

The study was conducted in compliance with the ethical guidelines of the Health Research Ethics Committee of the University of Nigeria Teaching Hospital, Enugu after obtaining ethical approval (reference number; UNTH/CSA/329/OL.5). Written informed consent was obtained from the respondents after they had been duly informed of the purpose of the study and assured of the confidentiality of volunteered information.

### Study area, study design and study population

Enugu state is one of the five states in the southeast geo-political zone of Nigeria. It is made up of seventeen local government areas, (LGAs) that are endemic to onchocerciasis. [15] The implementation of community-directed Treatment with Ivermectin strategy by the African Programme for Onchocerciasis Control (APOC) was initiated in the state in 1999. Since then, ivermectin is administered orally at a dose of 150μg/kg yearly to all the community members in the State by the community-directed distributors as prophylaxis for onchocerciasis. [15]

The study was a community-based cross-sectional comparative study conducted between September 2019 and November 2019. The study populations were onchocerciasis patients and healthy subjects living in the same neighbourhood in Enugu State, Southeast Nigeria. For the respondents with onchocerciasis, inclusion criteria included being 18 years and above with observable onchocerciasis manifestations that were identified through physical examination. The controls selected were age- and gender-matched respondents without onchocerciasis.

### Case definition and diagnosis

Onchocerciasis was defined as any person with fibrous nodules in the subcutaneous tissues, dermatitis, depigmentation of the skin (Leopard skin), or hanging groin.[7,16] The Diagnosis of onchocerciasis was obtained through physical examinations of the respondents by the health care providers that work with the State Ministry of health, Onchocerciaisis unit to ascertain those with onchocerciasis-related skin manifestations.

### Sample size determination

The sample size was determined using the formula for comparing two independent proportions. [17] The prevalence of Onchocerciasis in Enugu state was taken as 27%, [15] with the estimated difference between the two study groups taken as 20%. A total of 340 respondents were recruited for each group based on type 1 error (α) of 0.05 in a two-sided test and power of 0.8.

### Sampling technique

A multistage sampling technique was used to select the respondents for the study. In the first stage, a simple random sampling technique by balloting was used to select 7 local government areas out of 17 LGAs. In the second stage, from a list of all the wards in the selected 7 LGAs, two wards were selected in each LGA using a simple random sampling technique through balloting. Thus, fourteen wards were selected in the 7 local government areas. In the selected wards, consecutive recruitment of persons with onchocerciasis was done. This was achieved with the assistance of health workers in the onchocerciasis unit of the State Ministry of Health and those in the selected LGAs. The age and sex-matched healthy respondents were recruited from the same household or house as those with onchocerciasis. Where no one is qualified as control, the next house is examined for eligible control. The aim is to ensure that the cases and the controls were exposed to the same environmental factors.

### The study instruments

The SF-36 Health Survey is a generic, multi-purpose, short-form health survey that contains 36 variables for assessing the health-related quality of life. [18] The 36-item questionnaire is used to calculate eight sub-domains including physical functioning, role limitation due to physical health, bodily pain, general health, energy/fatigue, social functioning, role limitation due to emotional problems, and emotional well-being. [18,19] The scores for the first four sub-domains could be summed to create the physical composite score (PCS), while the last four sub-domains form the mental composite score (MCS), These two composite scores are the two major domains. The combination of the physical composite score (PCS) and mental composite score (MCS) gives the Overall quality score (HRQoL score). All questions are scored on a scale from 0 to 100, with 100 representing the best health status and 0 indicating the worst health status. These scores (0–100) were assigned for each subdomain and domain. For the overall quality scores, the respondents were categorized into good and poor quality of life; the proportion of respondents that scored <50% was classified as having poor quality of life while those that had ≥50% were categorized as having a good quality of life.

WHO Disability Assessment Schedule 2.0 (WHODAS 2.0) is a 36-item questionnaire that was used to assess the level of disability for people with onchocerciasis only. [20] Their disability scores were weighted as 1 = none, 2 = mild, 3 = moderate, 4 = severe, and 5 = extreme. For the overall disability scores, the weighted scores were converted to 1 = 20%, 2 = 40%, 3 = 60%, 4 = 80%, 5 = 100% and the respondents were categorized into no disability and some disability when they scored <50% and ≥50% respectively.[20] Also, the WHO Model Disability Survey (MDS) questionnaire[21] was adapted and used to assess the perception of the attitude of society towards people affected with onchocerciasis and perception of being a burden to the people. The overall perception of the attitude of society towards people with onchocerciasis was assessed using five variables which were; problem in participating in the family decision, problem in making a big decision in life, a problem with getting involved in society because of attitude, a problem with getting accepted by people and problem in being respected by people. Each of the 5 variables was given a score of 1 for a correct response and 0 for an incorrect response. The overall perception was categorized into poor perception for scores <50% and ≥50% for good perception. The perception of being a burden to the people was assessed by “Yes” or “No” response to one variable which is whether the respondent considers himself/herself as a burden to others.

Information was also obtained on the socio-demographic characteristics of the respondents and the socio-economic class.

### Data analysis

Data entry and analysis were done using IBM Statistical Package for Social Sciences (SPSS), version 25. Continuous variables were summarized using mean and standard deviation, while categorical variables were summarized using frequencies and proportions. The outcome measure of the study was health-related quality of life. The eight specific sub-domains, the physical composite score and mental composite score were summarized using mean and standard deviation. These variables were compared between respondents in study and comparison groups using the Student t-test. The proportion of respondents in the study and comparison groups who had a poor quality of life was compared using the Chi-square test of statistical significance. The Chi-square test was used to measure associations between independent variables and health-related quality of life. Variables with a p-value <0.2 at Chi-square analysis were subjected to logistic regression for multivariable analysis to determine predictors of poor quality of life. Correlation analysis was performed between total disability scores and total quality scores of the people infected with onchocerciasis. The level of statistical significance was set at a p-value <0.05 for all inferential analyses; The results of the multivariable logistic regression analysis were presented using an adjusted odds ratio and 95% confidence interval.

The socio-economic status index was developed using Principal Component Analysis, (PCA) and this was obtained for individuals infected with onchocerciasis only. The input to the PCA included information on the type of housing, ownership of fifteen household items that included having electric power, radio, refrigerator, functional car, television, mobile phone, gas cooker, bicycle, kerosene, generator, rechargeable lamp, electric iron, electric fan and air conditioner, the principal type of toilet facility, the main source of energy, household consumption of food and non-food items and average monthly income of respondent and that of his/her spouse where applicable. [22] These were used to develop the wealth index according to quartiles. The quartiles included Q1 = Poorest, Q2 = The Very Poor, Q3 = The Poor, and Q4 = The Least Poor. The quartiles were further dichotomized into low socio-economic class comprising the poorest and very poor and high socio-economic class made up of the poor and least poor groups.

## Results

### Socio-demographic characteristics

Table 1 shows the socio-demographic characteristics of the respondents. The mean age of respondents in the study group, 46.8±17.5 years was comparable to that in the control group, 45.8 ±16.5 years, (Student t = 0.685, p = 0.494). Regarding the level of education in both groups, 37.1% of the study group and 57.1% of the control group had secondary education as their highest level of education which was statistically significant (χ2 = 38.986, p = <0.001). The highest proportion of respondents in the two study groups were self-employed, (Study group, 64.4%; Control, 68.5%) while the least proportion was on salaried employment (Study, 16.2%; Control, 11.5%) but the difference in proportions was not found to be statistically significant, (χ^2^ = 3.182, p = 0.203)

**Table 1:** Socio-demographic characteristics of the respondents.

Variable	Study Group (n = 340) N (%)	Control Group (n = 340) N (%)	χ^2^ (P-value)
**Age of respondents**			
Mean (±SD)	46.8±17.5	45.8 ±16.5	0.685** (0.494)
**Age of respondents in groups**			
<35 years	96 (28.2)	96 (28.2)	0.011 (1.0)
35–44 years	54 (15.9)	54 (15.9)	
45–54 years	69 (20.3)	70 (20.6)	
≥55 years	121 (35.6)	120 (35.3)	
**Gender**			
Male	141 (41.5)	141 (41.5)	FT* (1.0)
Female	199 (58.5)	199 (58.5)	
**Educational attainment**			
No formal education	66 (19.4).	26 (7.6)	38.986 (<0.001)
Primary education	89 (26.6)	57 (16.8)	
Secondary education	126 (37.1)	194 (57.1)	
Tertiary education	59 (17.4)	63 (18.5)	
**Marital status**			
Never married	81 (23.8)	114 (33.6)	FT* (<0.001)
Married	242 (71.2)	224 (66.1)	
Separated/divorced	7 (2.1)	0 (0.0)	
Widowed	10 (2.9)	1 (0.3)	
**Employment status**			
Unemployed	66 (19.4)	68 (20.0)	3.187 (0.203)
Self-employed	219 (64.4)	233 (68.5)	
Salaried employment	55 (16.2)	39 (11.5)	

*Fischers exact test

**Student t

### The sub-domains of the Health-related quality of life

The sub-domain mean scores of people with onchocerciasis were significantly lower than the healthy population (p<0.001). Also, the PCS and MCS of the respondents with onchocerciasis were 64.6±24.8 and 64.3±20.7, which were lower than those of the controls (81.1±16.6 and 78.1±13.6), *p* < 0.001, as shown in Table 2.

**Table 2:** Summary of HRQOL Measures in all the eight (8) sub-domains and composite scores among the respondents in study and control groups.

Variables	Study group (n = 340) Mean±SD	Control group (n = 340) Mean±SD	Student t test	P-value
Physical functioning total score	72.1±29.7	90.4±14.4	10.209	<0.001
Role limitation in physical	56.8±45.9	84.4±31.8	9.137	<0.001
Role limitation in emotional health	58.7±46.1	84.9±32.9	8.525	<0.001
Energy/fatigue	60.1±18.2	71.1±13	8.882	<0.001
Emotional well-being	68.7±16.5	74.0±14.0	4.521	<0.001
Social functioning	69.5±24.1	82.2±17.8	7.850	<0.001
Pain	66.2±26.8	77.0±22.7	5.665	<0.001
General health	63.3±21.0	72.3±18.3	5.986	<0.001
Physical component summary (PCS)	64.6±24.8	81.1±16.6	10.167	<0.001
Mental component summary (MCS)	64.3±20.7	78.1±13.6	10.230	<0.001
Overall score	64.5±21.9	79.6±13.2	10.642	<0.001

### Quality of life among the respondents

**Fig 1:**
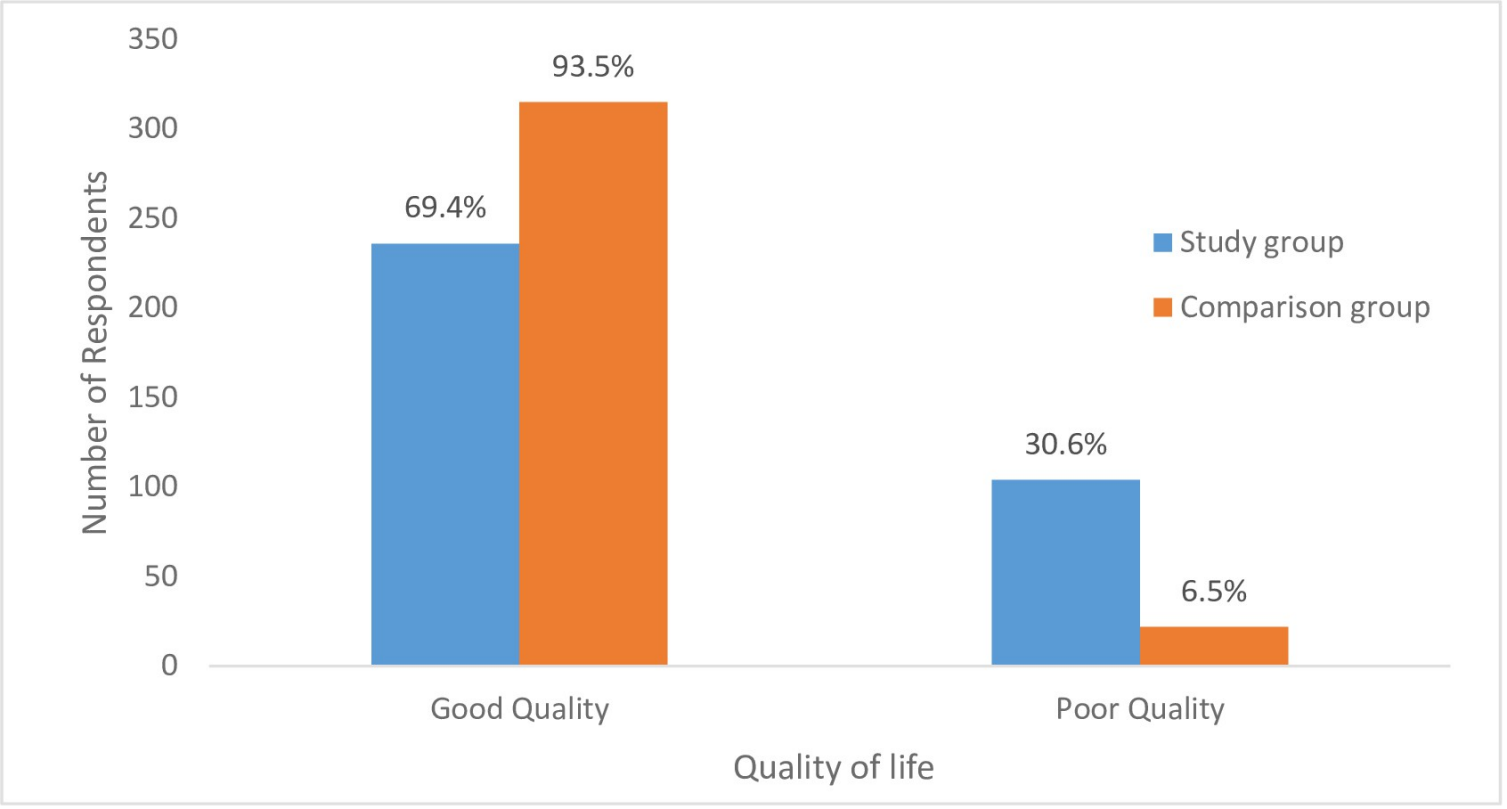
Comparing overall Health-Related Quality of Life measures among respondents with onchocerciasis and the control group.

Fig 1 shows the quality of life among respondents with onchocerciasis and the control group. A higher proportion of respondents in the control group, 93.5% had a good quality of life when compared with onchocerciasis patients, 69.4% and the difference in proportions was found to be statistically significant, (χ2 = 64.680, p<0.001)

### Perception of the attitude of others towards respondents with onchocerciasis

A greater proportion of the respondents with onchocerciasis reported that they had no problems in participating in the family decision, making a big decision in life, getting involved in society, getting accepted by people and being respected. Also, the majority of the respondents reported a good perception of the attitude of others towards them compared to those who had a poor perception of them. (Table 3)

**Table 3:** Perception of the attitude of others towards respondents with onchocerciasis.

Variables	Frequency (n = 340)	Percent (%)
A problem in participating in family decision		
No (correct)	273	80.3
Yes	67	19.7
Problem making a big decision in life		
No (correct)	270	79.4
Yes	70	20.6
Problem getting involved in society because of attitude		
No (correct)	249	73.2
Yes	91	26.8
Problem getting accepted by people		
No (correct)	275	80.9
Yes	65	19.1
A problem in being respected by people		
No (correct)	267	78.5
Yes	73	21.5
Overall perceptions of the attitude of others towards respondents		
Good	268	78.8
Poor	72	21.2

### Relationship between quality of life scores and disability scores for persons with onchocerciasis

**Fig 2:**
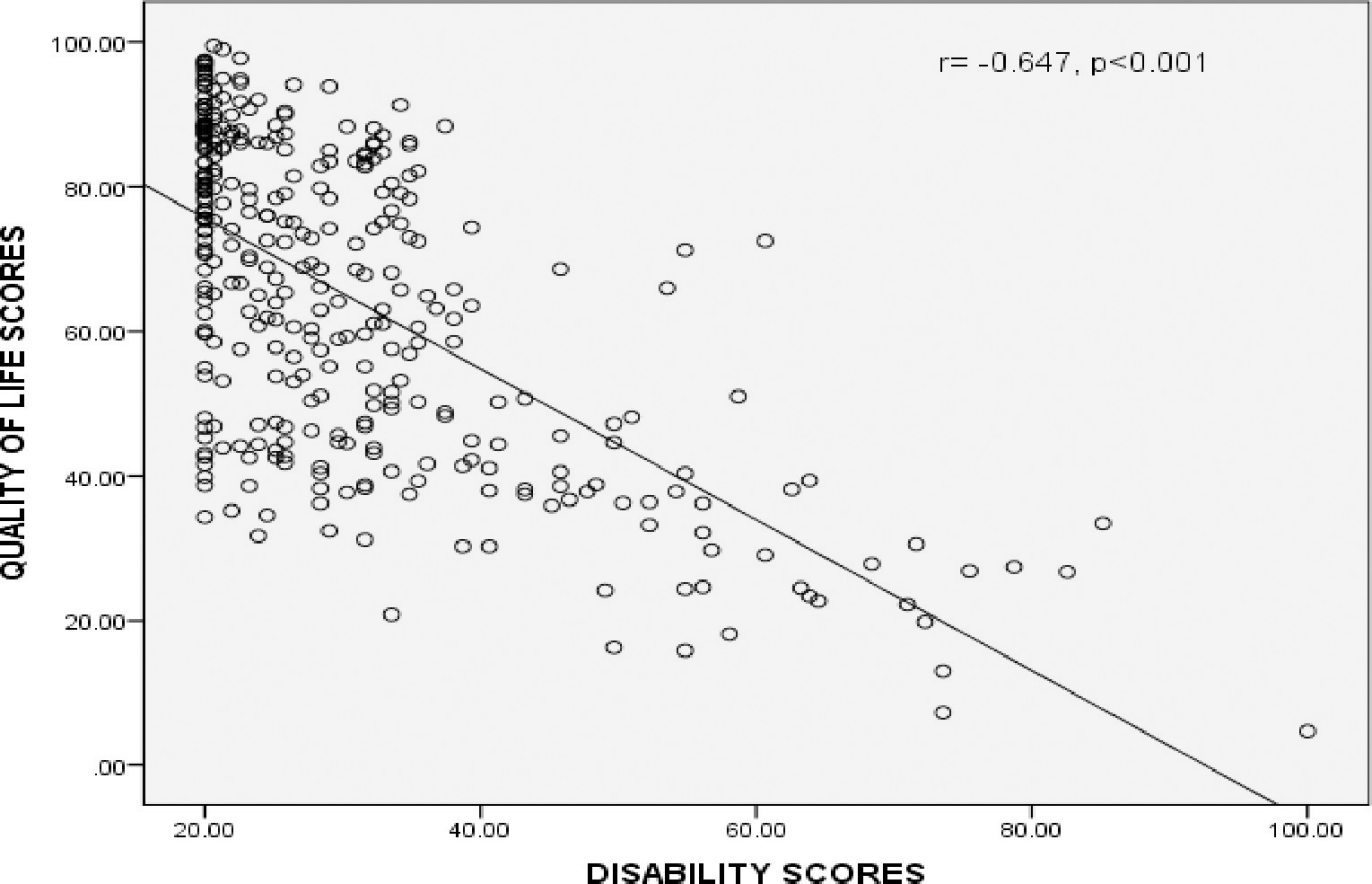
Correlation of total Quality of life scores with total disability scores among respondents with onchocerciasis.

Table 4 shows the correlation matrix for respondents with onchocerciasis. Among the onchocerciasis patients, there was a strong negative correlation between the total quality of life scores and total disability scores, increases in disability scores correlated with decreases in quality of life and this was found to be statistically significant, (n = 340, r = -0.647, p<0.001). (Fig 2).

**Table 4:** Correlation matrix for persons with onchocerciasis.

Variable	(n = 340) Correlation coefficient r, (p value)		
	Total quality of life scores	Total disability score	Age in years
Total quality of life scores			
Total disability score	r = -0.647 p<0.001		
Age in years	r = - 0.516 p<0.001	r = 0.471 p<0.001	

### Factors affecting the poor quality of life among respondents with onchocerciasis

In Table 5, The variables (age, educational attainment, marital status, disability, socioeconomic class, employment status, perception of attitude to others, perception of being a burden to people) with a p-value <0.2 at bivariate analysis were fitted to a multivariate logistic regression model to determine predictors of poor quality of life. The analysis showed that age, disability and perception of being a burden to people were significant predictors of poor quality of life. The respondents with onchocerciasis aged 48 years and above were about 3 times more likely to have a poor quality of life when compared with those aged <48 years. (AOR = 2.5; 95%CI: 1.4–5.0). The respondents with some disability were about three times more likely to have a poor quality of life when compared with those who had no disability.(AOR = 3.33;95%CI:1.4–5.0). Respondents who perceive themselves as a burden to others were about ten times more likely to have a poor quality of life compared to those who don’t perceive themselves as a burden to others. (AOR = 10; 95%CI: 2.5–20).

**Table 5:** Bivariate and multivariate analyses on factors affecting the poor quality of life in the respondents with onchocerciasis.

Variable	Health-related quality of life (n = 340) N(%) N(%) Poor Good		χ^2^ (p-value on bivariate analysis)	AOR in multivariable analysis (95% CI)
**Age of respondents**				
≥48 years	77(47.0)	87(53.0)	39.953(<0.001)	2.5 (1.4–5.0)
<48 years	27 (15.3)	149 (84.7)		1
**Gender**				
Male	42 (29.8)	99 (70.2)	0.073 (0.787)	NA
Female	62 (31.2)	137 (68.8)		
**Educational attainment**				
No formal education	32 (48.5)	34 (51.5)	17.306(<0.001)	1.1 (0.4–3.3)
Primary education	30 (33.7)	59 (66.3)		0.9 (0.3–2.5)
Secondary education	25 (19.8)	101 (80.2)		0.5 (0.2–1.1)
Tertiary education	17 (28.8)	42 (71.2)		1
**Marital status**				
Married	80 (32.1)	162 (66.9)	2.412(0.120)	1.1 (0.5–2.0)
Single**	24 (24.5)	74 (75.5)		1
**Disability**				
Some disability	95(36.7)	164(63.3)	18.999(<0.001)	3.33 (1.4–5.0)
No disability	9 (11.1)	72 (88.9)		1
**Socioeconomic class**				
Low socioeconomic class	70 (41.2)	100 (58.8)	17.953(<0.001)	1.7 (0.8–3.3)
High socioeconomic class	34 (20.0)	136 (80.0)		1
**Employment status**				
Unemployed	14 (21.2)	52 (78.8)	4.398(0.111)	0.6 (0.2–1.7)
Self-employed	75 (34.2)	144 (65.8)		0.7 (0.3–1.7)
Salaried employed	15 (27.3)	40 (72.7)		1
**Perception of attitude to others**				
Good	76 (28.4)	192 (71.6)	2.964(0.085)	0.9 (0.5–1,7)
Poor	28 (38.9)	44 (61.1)		1
**Perception of being a burden to people**				
Yes	23 (76.7)	7 (23.3)	32.904(0.001)	10 (2.5–20)
No	81 (26.1)	229 (73.9)		1

**Never married, separated, divorced

## Discussion

The definitive goal in patients’ care is to restore or preserve functioning and general well-being related to the health condition especially among onchocerciasis patients who usually have debilitating and disfiguring clinical manifestations of the disease that can affect their quality of life. [6,23]

The assessment of the health-related quality of life among persons with onchocerciasis and the comparison group shows a significant difference in the health-related quality of life between the groups. People with onchocerciasis had significantly lower mean HRQOL scores, compared to the comparison group or non-ill respondents in all the eight domains but role limitations in the physical and emotional domain were more affected. The highest HRQOL mean score in this study was found in the physical function domain probably due to the less debilitating effect of onchocerciasis on the musculoskeletal systems, compared to other NTDs like lymphatic filariasis and podoconiosis. [11,24] Also, the mean physical and mental component summary scores of respondents, in the study group, were significantly lower than in the comparison group. These findings of lower HRQOL mean scores were similar to reports in other NTDs from previous researchers. [11,25,26] The selection of controls from the same household as the study group may predictively underestimate differences between the two groups due to the likely impact of onchocerciasis on the health, economic and social well-being of the entire household. Despite this expected modulatory effect, this study found a significantly lower HRQoL in all the domains in people with onchocerciasis which implies that onchocerciasis interferes generally with quality of life, especially, with the ability to carry out normal physical and mental functions, probably due to the negative impact of the disease on the body and the psychological stress associated with the disease. [24,27]

Regarding the overall health-related quality of life, a significantly higher proportion of respondents with onchocerciasis had poor quality of life when compared with those in the control group. This was consistent with other studies done among NTDs patients (lymphatic filariasis, podoconiosis and leprosy). [11,24,26,28]The differences in the proportions between those with onchocerciasis and the healthy population could probably be due to the differential effects of onchocercal lesions on the respondents in the study group, such as the various dimension of skin lesions that interfere with their daily life activities like self-care, ability to earn a living and other life roles in family and society. [6,7]

In this study, the majority of the respondents perceived that family and society had a good and encouraging attitude towards them, and this acceptance may help improve their quality of life. However, this disagrees with the findings from Edo State, Nigeria. [29] The lower societal attitude documented by Wagbatsoma et al [29] could be due to their inclusion of onchocercal eye lesions as these lesions are noted to be more debilitating compared to skin lesions. Furthermore, in this study, the favorable perception of the society or the non-stigmatizing attitude towards onchocerciasis patients could be as a result of better management strategy for the disease entity, understanding of the etiology of the disease and better awareness creation on its inability to be transmitted from one man to another. [30] All these seem to have caused a paradigm shift in attitude and perception of people, with a positive effect on HRQOL. [3,6]

A strong inverse relationship was noted between disability due to onchocerciasis and quality of life. Thus an increase in disability causes a significant negative impact on the HRQOL which is consistent with a previous report. [31] This may be explained by the fact that onchocerciasis causes debilitating clinical features which range from mild skin itching to severe skin lesions but their effects in causing activity limitations and disability differs. [32,33] People who have debilitating skin manifestations may tend to lag in terms of schooling and employment, and this may affect their general well-being leading to more reduction in quality of life compared to others with mild disabilities such as skin itching. [32] This has put onchocerciasis on the front burner of public health discourse, as one of the leading causes of disability, since it affects not just their health, but limits their ability to fulfill societal roles, thereby preventing people from living a good quality life. [6,34]

The respondents who were 48 years and above were about 3 times more likely to have a poor quality of life when compared with those aged below 48 years. This corresponds with a study in Brazil and the probable explanation to the finding may be that aging is associated with chronic degenerative diseases which limits mobility and daily life activities, and living with onchocerciasis infection may worsen their situation. [35] This implies that the majority of the aged persons with onchocerciasis may not be able to take care of themselves or carry out some of the daily life activities making them a burden to their families and society.

Also, respondents with some disability were about three times more likely to have a poor quality of life when compared with those who had no disability. Thus the more disabled an individual becomes, the more the quality of life could be compromised and this is similar to the finding from another study. [31] Respondents who do perceive themselves as a burden to others were ten times more likely to have a poor quality of life compared to those who do not perceive themselves as a burden to others. However, there were dissimilarities with findings from other NTDs such as podoconiosis, leprosy, and lymphatic filariasis. [11,24,36] These diseases are highly debilitating, stigmatizing, and the individuals are unable to take care of themselves which makes them a burden to the family and society. [24,36] The possible explanation for the findings of this study may be that those who don’t consider themselves a burden to the family nor the society, easily interact with other people and perform their societal roles thus, were not consumed in self-pity or stigmatize self. This positive attitude could reduce depression and other psychological problems associated with onchocerciasis, with consequent improvement of quality of life.

The limitation of this study is that the information obtained from the respondents was subjective because of the possibility of recall bias and the self-reporting nature of the study instrument (HRQoL). This could have been reduced by a complimenting qualitative method to obtain an in-depth understanding of the individual experiences regarding the quality of life. Another limitation of this study is the non-inclusion of individuals with onchocercal eye lesions. This may affect the disability and quality of life findings among respondents with onchocerciasis.

## Conclusion

People with onchocerciasis experience a lower health-related quality of life compared to the healthy population and this strongly links to disability. The influencing factors of HRQoL in onchocerciasis are age, disability and perception of being a burden. Therefore, the stakeholders in the onchocerciasis program should increase community awareness on symptoms of onchocerciasis infection to ensure early detection and treatment to reduce disability and enhance HRQOL. Also, efforts to improve the quality of life of infected people should prioritize those with advanced disease and the aged.
